# Ascending aorta pseudoaneurysm 40 years following surgical atrial septal defect repair – A case report and literature review

**DOI:** 10.1177/02676591251408657

**Published:** 2025-12-20

**Authors:** Hong Jun Yong, Peter Lang, Ramanish Ravishankar, John Dreisbach, Gruschen Veldtman, Robyn Smith, Sukumaran Nair

**Affiliations:** 1 School of Medicine, Dentistry & Nursing, 3526 University of Glasgow, Glasgow, UK; 2 Department of Cardiothoracic Surgery, 568987 Golden Jubilee National Hospital, Glasgow, UK; 3 Department of Radiology, Golden Jubilee National Hospital NHS Trust, Glasgow, UK; 4 Scottish Adult Congenital Cardiac Service, 568987 Golden Jubilee National Hospital, Glasgow, UK; 5 Department of Anaesthesia and Intensive Care, 568987 Golden Jubilee National Hospital, Glasgow, UK

**Keywords:** ascending aortic pseudoaneurysm, atrial septal defect, cardioplegia cannula, surgical patch aortotomy, suturing technique

## Abstract

**Introduction:**

Ascending aortic pseudoaneurysm is a rare postoperative complication of cardiac surgery, often linked to infection and dehiscence at sites of aortotomy, cannulation, cross-clamp sites, and proximal coronary anastomoses.

**Case Report:**

We present a case of an ascending aortic pseudoaneurysm in 48-year-old woman who underwent atrial septal defect repair at 8 years old. Based on pre-operative imaging and intra-operative findings, the pseudoaneurysm developed from previous cardioplegia cannula site. She underwent emergency surgery where the pseudoaneurysm was resected and repaired with a Gelweave patch. The patient recovered well with no post-operative chest pain or dyspnoea at 5-months follow-up.

**Conclusion:**

Cardioplegia cannula remains a rare but significant iatrogenic cause of aortic pseudoaneurysm. Prevention of pseudoaneurysm formation can be achieved by inserting the cardioplegia cannula tip through full-thickness purse-strings and/or addition of separate oversewing sutures after decannulation during the primary operation.

## Introduction

Ascending aortic pseudoaneurysms that occur as a complication of cardiac surgery are rare and are often attributed to infection and dehiscence of aortic suture lines, cannulation sites, cross-clamp sites, and bypass graft sites.^1,2^ We report a case of an ascending aortic pseudoaneurysm of the aortic cardioplegia cannula insertion during previous ASD repair, performed 40 years ago.

## Case report

A 48-year-old woman with a background of a surgically repaired atrial septal defect (ASD) at 8 years old was referred for emergency surgical repair of ascending aortic pseudoaneurysm. She initially presented with an acute history of central chest pain, with pain on palpation of the costal margins bilaterally and orthopnoea. Due to a raised D-dimer, a computed tomography pulmonary angiography (CTPA) was performed. This showed no evidence of pulmonary thromboembolism but revealed a calcified outpouching with internal thrombus, adjacent to the right atrium (RA).

Subsequent CT coronary angiography (CTCA) revealed a 21 mm × 10 mm x 12 mm pseudoaneurysm arising anteriorly from the proximal ascending aorta, 6 mm superior to the ostium of the right coronary artery (RCA) and 5 mm above the Sino-tubular junction (STJ) (Figure 1(a)). Intracavitary thrombus and active blood flow through the pseudoaneurysm was also noted. The pseudoaneurysm sac was heavily calcified, except for its inferior aspect, where the wall was thin and vulnerable to rupture.

**Figure 1.**
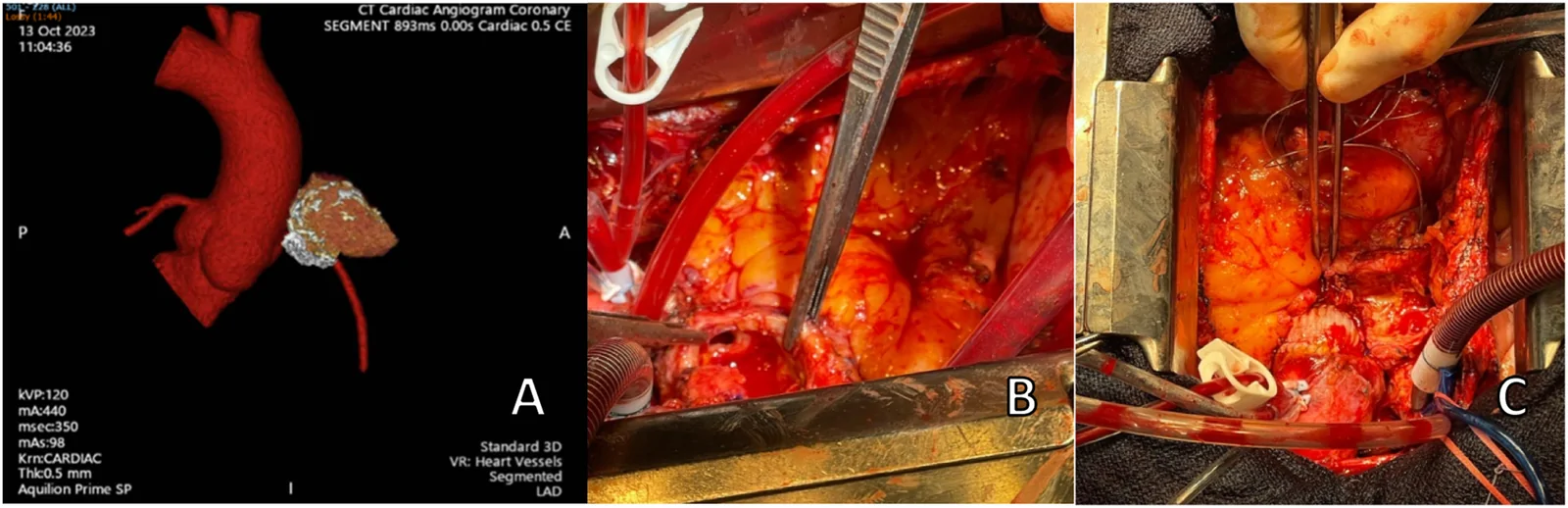
(a) Three-dimensional reconstruction of CTCA shows a 21 mm × 10 mm x 12 mm pseudoaneurysm (*white arrow*), arising anteriorly from the proximal ascending aorta. The pseudoaneurysm sac is heavily calcified, save for its inferior aspect where the wall is thin and vulnerable to rupture. (b) Intraoperative image showing a 1-cm diameter defect (*white arrow*) on the right side, anterolaterally above the sinotubular junction connecting the pseudoaneurysm sac to the main aorta. Likely site of cardioplegia cannulation in previous cardiac surgery. (c) Intraoperative image shows reconstruction of ascending aorta at site of pseudoaneursym using an elliptical Gelweave patch.

Due to the location of the pseudoaneurysm, cardiopulmonary bypass was initiated via peripheral cannulation of the right common femoral vessels. Redo sternotomy was performed using a redo circular oscillating saw. Antegrade cardioplegia was used through an aortic root vent. In keeping with the pre-operative CTCA findings, the pseudoaneurysm sac was heavily calcified anteriorly and thin walled inferiorly. Consistent with site of previous cardioplegia cannula site during ASD repair 40 years ago, the sac was located above the STJ, arising from the anterolateral part of the proximal ascending aorta on the right side, approximately 6 mm above the RCA ostium (Figure 1(b)). Incision into sac revealed intracavitary thrombus and a 1 cm defect connecting the aorta to the pseudoaneurysm was located on the right anterolateral aspect of the ascending aorta, directly above the RCA ostium (Figure 1(b)). Despite the proximity, the pseudoaneurysm sac did not impinge on the RCA ostium. Following excision, the aortic defect was non-circumferential and therefore amenable with patch repair.

An elliptical Gelweave^TM^ patch (Vascutek Ltd, Inchinnan, Renfrewshire, UK) was fashioned from a standard tube graft to match the size of the defect and sutured over the defect using a continuous 4/0 polypropylene sutures. (Figure 1(c)). BioGlue® surgical adhesive (CryoLife Inc., Georgia, USA) was then applied externally to the suture line. Cross-clamp time was 145 min, and CPB time was 160 min. Following careful cardioplegia decannulation, full thickness stay sutures were tied. Post-CPB TOE showed good biventricular and valve function.

Post-operative recovery was uneventful with no noted neurological deficits. The patient was discharged on post-operative day 7 with warfarin for 3 months, aiming for target INR 2.5-3.5. At 5 months follow-up, the patient reported no further symptoms.

## Discussion

Ascending aortic aneurysms are a rare post-operative complication of cardiac surgery, occurring in <0.5% of cases.^3^ Most reported pseudoaneurysms result from infection or suture dehiscence at site of aortotomy, aortic cross-clamp, aortic cannulation, proximal coronary anastomoses, or prior aortic surgery sites.^1,2^ We report one of the few cases of pseudoaneurysms occurring at the cardioplegia cannula site,^4^ highlighting that pseudoaneurysms can arise from any surgical manipulation point and at any timeframe post-operatively— with one study reporting 37 years between primary surgery and pseudoaneurysm repair.^5^ Early surgical intervention is essential due to rupture risk and high morbidity and mortality rates between 29 and 46%; often due to rupture upon sternal re-entry or surgical manipulation.^6,7^

Despite its rarity, it is important to consider the aetiologies of pseudoaneurysm to ensure appropriate practises are conducted to avoid this complication. In addition to the risk factors of pseudoaneurysm, poor suture technique and incautious aortic manipulation can also increase the risk of its occurrence. Although the cannulation technique of initial procedure is not known, historical data suggests this would conclude with single or double purse strings without pledgets. In our case, we recommend extra precautions should be taken whilst inserting the cardioplegia cannula tip through full-thickness purse-strings, adding separate oversewing sutures and oversewing with pledgets to reinforce aortic tissue after decannulation.

The keystone of pseudoaneurysm repair is to prevent fatal haemorrhage and ensure cerebral protection^7^ by considering cannulation strategy and hypothermia. Due to likely retrosternal adhesions, we used femoral-femoral bypass to decompress the heart for safer sternal re-entry. As the pseudoaneurysm was <55mm—the typical rupture size on re-entry—we initiated CPB just before cross-clamp.^8^

For our case, aortic cross-clamping was possible on the distal ascending aorta; subsequently the pseudoaneurysm was excised and viable aortic tissue was noted. Both surgical patch aortotomy and aortic graft interpositions have significant survival benefit, and either strategies adopted for pseudoaneurysm repair are effective.^9^ Selection of technique is dependent on its anatomy, namely its size and degree of native aortic wall involvement. We recommend the use of surgical patch aortotomy if there is salvageable aortic tissue after pseudoaneurysm excision, in order to minimise manipulation and preserve native tissue. Native aortic tissue shows greater extensibility and more effective diastolic pump function in comparison to graft material, thus preserving the normal efficiency of the aortic root complex and reducing the risk of long-term graft failure.^10^

## Conclusion

Aortic pseudoaneurysm is a rare complication, requiring early diagnosis and prompt surgical intervention. This case highlights the importance of minimizing aortic wall injury to reduce the risk of pseudoaneurysm formation.
